# Development and test of a visual-only meat inspection system for heavy pigs in Northern Italy

**DOI:** 10.1186/s12917-017-1329-4

**Published:** 2018-01-05

**Authors:** Sergio Ghidini, Emanuela Zanardi, Pierluigi Aldo Di Ciccio, Silvio Borrello, Giancarlo Belluzi, Sarah Guizzardi, Adriana Ianieri

**Affiliations:** 1 Department of Food and Drug, Parma University, Via Del Taglio, 10, 43126 Parma, Italy; 2 Italian Ministry of Health, Via Giorgio Ribotta, 5, 00144 Rome, Italy; 3 Italian Ministry of Health, Viale Tanara 31/A, 43100 Parma (PR), Italy

## Abstract

**Background:**

There is a general consensus in recognizing that traditional meat inspection is no longer able to address the hazards related to meat consumption. Moreover, it has been shown that invasive procedures, such as palpation and incision, can increase microbial contamination in carcasses. For these reasons, legislations all over the world are changing meat inspection techniques, moving towards visual-only techniques. Hence, there was also the need to test visual-only inspection in pigs in Italy.

**Results:**

A protocol for visual-only post-mortem inspection was produced together with a 24-class scheme used to record pathological lesions. A list of guidelines needed for univocal interpretation and classification of lesions was developed. To record lesions at the slaughtering line, a light instrument that is resistant to the slaughter environment was designed and then produced in collaboration with an electro-medical company. Six contracted veterinarians were chosen and trained. They performed visual-only post-mortem inspections on 231.673 heavy pigs in three different slaughterhouses of Northern Italy. Visual-only inspection was compared to traditional inspection on 38.819 pig carcasses. No relevant differences were found between the two systems.

**Conclusions:**

The comparison between traditional and visual-only inspection showed that visual-only inspection can be adopted in pig slaughterhouse. The analysis of the performance of the veterinarians stressed the importance of standardization and continuous education for veterinarians working in this field.

## Background

Veterinary inspection has been performed for more than a century in slaughterhouses, and it has been effective in protecting consumers against classical hazards such as *Mycobacterium bovis* and parasites. However, there is a consensus around the idea that traditional inspection methods in slaughterhouses no longer cope with the hazards that pose the highest foodborne risks today, such as Salmonella and Yesinia. In industrialised countries, classical diseases are now more effectively controlled with eradication plans [1]. Back in 2011, EFSA [2] stated that the traditional inspection system in swine is not targeted to the main hazards deriving from meat consumption. These hazards are no longer detectable by classical meat inspection because they are no longer caused by pathogens associated with specific lesions and are sometimes related to chemicals. Moreover, procedures such as palpation and incision of the viscera by veterinarians can lead to cross contamination of the carcasses [3].

Considering this evidence, in 2014, the European Commission amended EU Regulation 854/2004 via EU Regulation 219 [4], which laid down specific rules for the organisation of official controls on products of animal origin intended for human consumption [5]. In particular, the regulation stated that starting in June 2014, post-mortem inspection in domestic swine should only be visual and that the official veterinarians shall proceed with additional post-mortem inspection procedures using incision and palpation of the carcass and offal when, in his or her opinion, clinical signs and lesions may indicate a possible risk to public health, animal health or animal welfare.

A classification of pig producers as a function of their risk level could help the official veterinarian choose the inspection method [6]. Such a classification should be possible using the food chain information (FCI) module. However, FCI proved to be inefficient in providing such information [7]. In fully integrated chains, it is certainly easier to get more information regarding the farm of origin. Such additional information can be useful for a classification of the farms based on risk.

In Italy, pork production shows a variety of organisational structures and farm size patterns. In 2012, the national pig population was approximately 8.600.000 animals (Eurostat). Southern Italy is characterized by a large number of small-scale farms and many low productivity slaughterhouses, producing a total of 5.700.000 carcasses per year (2012). The North of Italy, where approximately 9.300.000 carcasses are produced per year, is characterized by large-scale indoor intensive farms and high production slaughterhouses (up to 500 carcasses/h). A peculiar feature of swine production in the North of Italy is that there is a very high degree of integration between farmers and meat producers because the majority of swine production in this area processes Protected Designation of Origin products (PDOs). The animals, therefore, share the same genetics, breeding techniques, and feeding schemes, and they have to be born in the North of Italy. In addition, the weight and age of the animals are quite constant since they have to fulfil the requirements of the Parma Ham disciplinary of production. In fact, the animals have to be slaughtered at a minimum age of 9 months and usually weigh approximately 160 kg at the time of slaughtering, with a very small dispersion around the mean because there are economic penalties for lighter and heavier animals [8].

Given this scenario, pig production in the North of Italy can be considered almost fully integrated. Therefore, the holdings in which pigs are raised in this area are fully controlled. When categorizing the holdings according to the risks they pose to public health, they fall into a low-risk class. For this reason, it was considered feasible to test visual-only inspection in this area.

In Italy, there are no data on possible applications of a visual-only inspection system in pigs. In addition, consistent data on post-mortem lesions for pigs at the slaughterhouse are lacking. There have been some local projects in Northern Italy, but the obtained data are not homogenous and comparable. Moreover, in their review, Stark el al. [9] highlighted “a substantial lack of suitable and accessible published data on the frequency of occurrence of many diseases and conditions affecting food animals in Europe.” In this context, the Italian Ministry of Health, on behalf of the National Committee for Food Safety, financed a project to study new inspection systems for both the South and the North of Italy.

To fulfil the needs of the high productivity slaughterhouses of the North of Italy, which are characterized by a high working speed, a visual-only inspection system was designed. The system was then tested in three slaughterhouses in the North of Italy to obtain data on the prevalence of post-mortem lesions in pigs dedicated to the production of PDO products. The visual system was then compared to the “traditional” inspection using invasive procedures.

## Methods

### Study area and population

The Parma Ham Consortium of production limits the area of origin of the animals dedicated to Parma Ham production (and other PDO products) to the following regions: Emilia-Romagna, Veneto, Lombardy, Piedmont, Molise, Umbria, Tuscany, Marche, Abruzzo and Lazio [8]. These regions represent the whole north and a large part of the centre of Italy. The pigs belong to the Large White, Landrace, Duroc breeds and their hybrids. They must be slaughtered at a minimum age of 9 months. At this age, they reach an average weight of 160 kg.

In 2013, 4199 farms in this area produced and then sent to slaughter 8.071.726 animals for transformation into PDO products. Pigs are usually sent to the slaughterhouses in batches of approximately 120 animals. The whole animals are slaughtered in 65 slaughterhouses. All the slaughterhouses have the possibility to buy animals from all the PDO regions mentioned above. Eighteen of these slaughterhouses, which are in only two regions (Emilia-Romagna and Lombardy), process 93% of all the animals [10]. For logistical convenience, the present study was performed in 3 of the 18 slaughterhouses (2 in Lombardy and one in Emilia-Romagna) that share the same layout and slaughtering technique and that are very similar in size and processing speed.

### Animal selection

Only heavy pigs following the Parma Ham disciplinary (therefore of national origin) were considered in this study. In the slaughterhouses, no further selection of the animals was performed so that all the animals of the Parma Ham area could have the same probability of being chosen for the study. To minimise the influence of the distance between the farm and the slaughterhouse, the sampling times were homogeneously distributed between the different working days of the week and the working hours of the day.

The study was designed to achieve relative standard errors of the prevalence of lesions lower than 1% for lesions with a prevalence higher than 5% and lower than 10% for lesions with a prevalence as low as 0.1%. Using FAO [11] formulae, we aimed to inspect 200.000 pig carcasses. The study lasted from January to August 2013.

### Visual inspection protocol

A new protocol of visual-only inspection for pigs was developed based on EU Regulation 854/2004 because there were no visual-only inspection protocols at the time of this study. To give an operative tool to veterinarians, the anatomical structures to be inspected were re-arranged into three main groups (carcass, red offal, green offal), which resembles the way organs are found at the end of a slaughtering line.

Together with the veterinary service in the Emilia-Romagna and Lombardy regions and the Italian Ministry of Health, a 24-class scheme (Table 1) was developed. The scheme was designed to be easily adopted in high production slaughterhouses, shared at national level and comparable with schemes adopted by the Food Safety and Inspection Service in the USA [12] and the Food Standard Agency in the UK [13]. A list of guidelines needed for univocal interpretation and classification of lesions was developed (Table 1).

**Table 1:** Lesion classification and the guidelines adopted to record the data

Apparatus	Lesion	Guideline
Respiratory	Pneumonia	Detect both pneumonia and outcomes of pneumonia. Detect pneumonia when an entire lobe is interested, or when not involving the entire lobe, it involves two contralateral lobes. Always consider specific pneumonia. Consider lung abscesses (even one) as pneumonia.
	Pleuropneumonia	Is recognized when adhesions are present on the carcass. Is recognised when fibrin is present on the visceral layer of the pleura.
Digestive	Hepatitis	Hepatitis and outcomes of hepatitis. The presence of fibrin on the capsule should not be classified as hepatitis (classified as peritonitis).
	Hepatosis/hepatic dystrophies	Steatosis and necrosis are to be classified only in cases involving at least an entire lobe or parts of several lobes.
	Peritonitis/perihepatitis	
	Enteritis	Haemorrhagic or necrotic. Thickening of the small intestine.
Reproductive-Urinary	Nephritis	Nephritis and glomerulonephritis.
	Nephrosis	Cystitis and hydronephrosis.
	Cryptorchidism	
Cardio Circulatory	Myocarditis	Involvement of pericarditis. Do not classify degenerative processes in the absence of inflammation as myocarditis.
	Pericarditis	
Integumentary	Dermatitis	Recognized when there is a thickening of the skin. Detect when lesions exceed 50% of the body surface and not when confined to the abdominal region and chest. Detect carcasses massively affected by bites of ectoparasites as dermatitis.
	Erysipelas	Detect whenever the typical skin lesions are encountered.
Locomotor	Arthritis	
	Muscle colour alteration (PSE/DFD)	PSE / DFD
	Oedema/emaciation	
Other (carcass)	Jaundice	
	Abscesses	Detect all abscesses that are not located in the lung or in the liver. Also detect phlegmons as abscesses.
	Neoplasms / tumours	
	Biliary or faecal contamination	Both faecal and bile contamination. In addition, the residual presence of parts of the rectal mucosa is considered contamination.
	Trauma	Skin
		Bruises and injuries due to mismanagement during loading / unloading (bruises and haematomas). Wounds from intraspecific fights and numerous injuries that get to in the derma, possibly infected.
		Skeletal muscle
		Splay-leg animals (open). Do not report results of old injuries.
	Lymphadenopathy	Mesenteric lymph nodes, lung, and generally an increase in the volume of lymph nodes in the carcass.
	Splenomegaly	Detect when affecting more than 50% of the organ.
	Petechial haemorrhages	

### Recording system

**Fig. 1:**
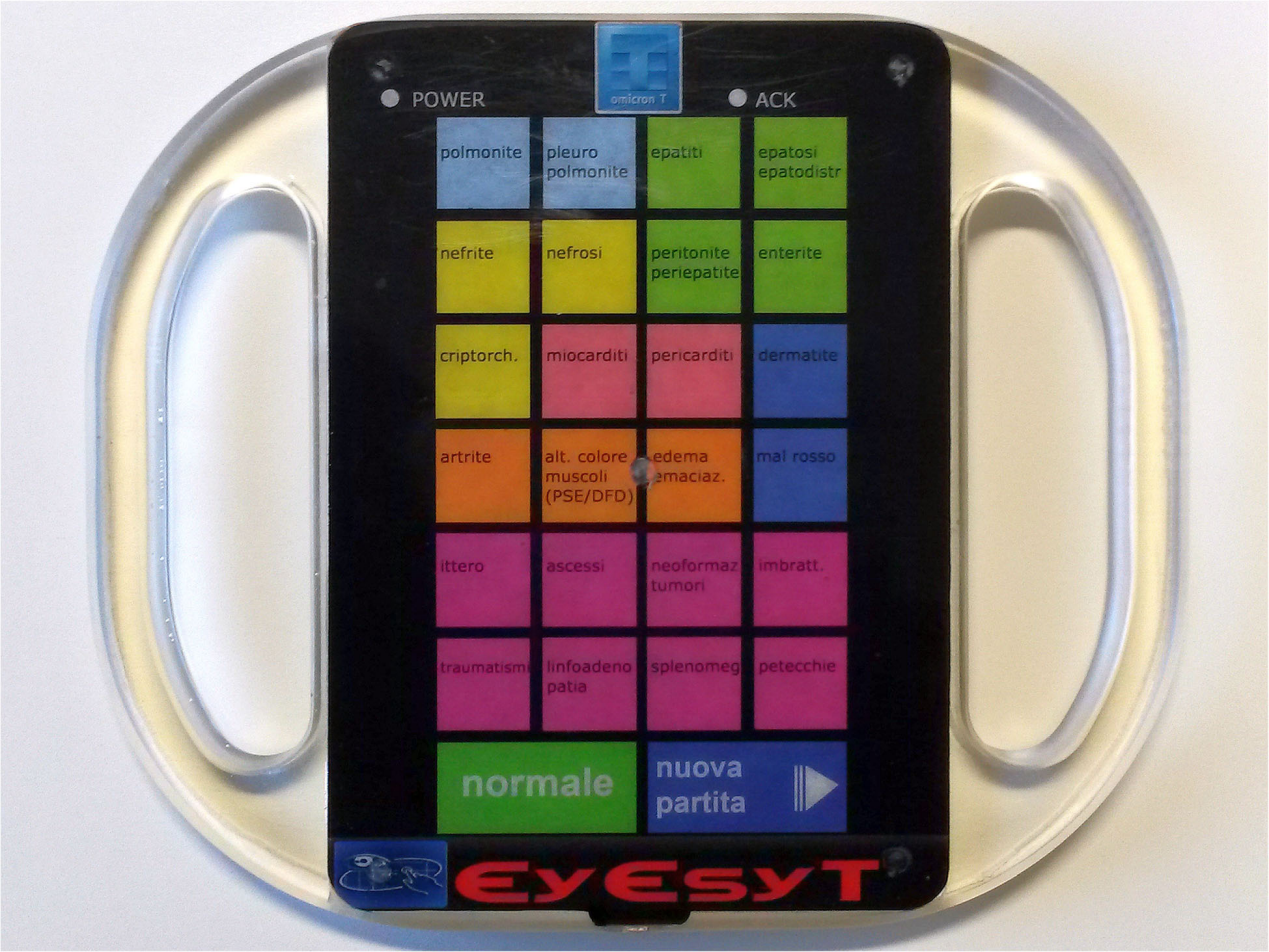
The recording system developed in cooperation with Omicron T

An electro-medical company (Omicron T S.R.L., Napoli, Italy) was commissioned to design a light tablet (Fig. 1). The tablet had to record lesions on the slaughtering line and be resistant to the slaughterhouse environment.

The instrument weights 420 g, and it is 24 cm wide, 25 cm height and 1 cm thick. It can be connected to a computer via a mini USB port, which is used for both data downloading and charging. On the front panel, it has 24 square buttons (2 cm on each side), representing the lesions in Table 1. Two larger buttons (2 cm high and 4 cm wide) are used for normal animals and to record a change of batch. A vibration is emitted when a button is pushed. In case of a mistake or a change in diagnosis, the operator can change his decision within 2 s, after which the decision is automatically confirmed by a flashing LED light. The data of each working day are then saved in a file and transferred to the central unit, which handles the database.

### Software and data analysis

Software was developed with the help of Omicron T S.R.L., (Napoli, Italy). The software had to build a database, starting from the data recorded on the tablets, and then handle a database of at least 400.000 inspected carcasses. The database system used by this software is MySql Server (Oracle, CA). At present, this software is able to extract the data from the database by using five filters: date, type of farm, distance from slaughterhouse, breeding farm code and veterinarian. In the future, the software could be implemented with other filters if necessary. The results of the queries were exported to MS Excel-compatible datasheets, and MS Excel was used for data elaboration. The mean data were compared using Student’s t-test.

### Personnel

Six veterinarians experienced in meat inspection of pigs were contracted to perform visual-only post-mortem inspection in the slaughterhouses. First, they were trained to use the recording system and then to handle it in operating conditions. Before collecting the data, each veterinarian was trained in the slaughterhouse for a period of about one month (approximately 5000 carcasses). After this period, their results were analysed and they were given further training on the classification of lesions, following the previously developed guidelines. The veterinarians then inspected approximately 40,000 carcasses each to achieve the target of 200,000 carcasses that was previously set. The contracted veterinarians were regularly rotated between the three slaughterhouses.

### Place of work

The three slaughterhouses had a capacity varying from 380 to 450 carcasses per hour. In these slaughterhouses, the contracted veterinarians were placed before the official colleagues performing traditional inspections to prevent the contracted veterinarians from diagnosing lesions by relying on cuts made by the official colleagues. To minimise mutual influence, the contracted and official veterinarians were always the maximum possible distance apart in the slaughtering environment (never <5 m) .

### Visual-only vs. traditional inspection comparison

In the last period of the study, the developed recording system was also given to official veterinarians, and the data from visual-only (performed by the contracted veterinarians) and traditional inspections (performed by the official veterinarians) of the same pigs were compared on 38.919 pig carcasses. In this period, the work was conducted only in one slaughterhouse to minimise environmental effects. Furthermore, because a different tool to record lesions was already in use in the chosen slaughterhouse, the official veterinarians working there were already trained to perform post-mortem inspections while recording data on an electronic device.

The study was submitted to the Institutional Review Board of The University of Parma that gave a favourable opinion since compliant with ethical principles.

## Results

Overall, 231.673 carcasses were inspected by means of a visual-only post-mortem inspection. The carcasses composed 1.832 batches (mean of 126 animals/batch) and came from 323 different farms. A batch is defined as a group of animals from one farm delivered on one day, usually transported by a single truck. In Table 2, the number and percentage of each lesion detected in each slaughterhouse and an estimate of the prevalence for each lesion. Table 3 presents the results of the comparison between traditional and visual-only inspections. Table 4 shows the total variability achieved and the variability within each lesion (standard deviation and variation coefficient).

**Table 2:** Number and percentage of each lesion detected during the work in the three slaughterhouses and an estimate of the prevalence of each lesion

.	Slaughterhouse 1		Slaughterhouse 2		Slaughterhouse 3		Tot.		
	number	%	number	%	number	%	number	Prevalence %	standard error
Pneumonia	5100	5.40	8840	8.99	1911	4.91	15,851	6.43	0.050
Pleuropneumonia	15,242	16.14	12,654	12.87	6756	17.35	34,652	15.46	0.074
Hepatitis	21,972	23.27	10,535	10.71	5594	14.37	38,101	16.12	0.075
Hepatosis/hepato-dystrophies	625	0.66	3537	3.60	618	1.59	4780	1.95	0.028
Peritonitis/perihepatitis	355	0.38	730	0.74	71	0.18	1156	0.43	0.013
Enteritis	206	0.22	514	0.52	137	0.35	857	0.36	0.012
Nephritis	234	0.25	261	0.27	113	0.29	608	0.27	0.011
Nephrosis	134	0.14	137	0.14	224	0.58	495	0.29	0.011
Cryptorchidism	139	0.15	140	0.14	115	0.30	394	0.20	0.009
Myocarditis	11	0.01	3	0.00	4	0.01	18	0.01	0.002
Pericarditis	3341	3.54	3345	3.40	1059	2.72	7745	3.22	0.036
Dermatitis	832	0.88	1120	1.14	858	2.20	2810	1.41	0.024
Erysipelas	29	0.03	115	0.12	291	0.75	435	0.30	0.011
Arthritis	0	0.00	8	0.01	0	0.00	8	0.00	0.000
Muscle colour alteration (PSE/DFD)	7	0.01	2	0.00	4	0.01	13	0.01	0.002
Oedema/emaciation	22	0.02	12	0.01	4	0.01	38	0.02	0.003
Jaundice	79	0.08	11	0.01	9	0.02	99	0.04	0.004
Abscesses	571	0.60	865	0.88	422	1.08	1858	0.86	0.019
Neoplasms / tumours	25	0.03	7	0.01	9	0.02	41	0.02	0.003
Biliary or faecal contamination	2126	2.25	3582	3.64	956	2.46	6664	2.78	0.034
Trauma	405	0.43	1316	1.34	674	1.73	2395	1.17	0.022
Lymphadenopathy	138	0.15	47	0.05	25	0.06	210	0.09	0.006
Splenomegaly	254	0.27	173	0.18	176	0.45	603	0.30	0.011
Petechial haemorrhages	24	0.03	1	0.00	0	0.00	25	0.01	0.002
Tot.	51,871	54.94	47,955	48.77	20,235	51.45	119,965	51.72	0.102
Animals	94,411		98,333		38,929		231,590

**Table 3:** Results of the comparison between traditional and visual-only inspections

	Traditional	%	Visual-only	%	δ % over traditional	Relative δ % over traditional
Pneumonia	2709	6.96	1911	4.91	−2.05	−29.5
Pleuropneumonia	4150	10.66	6756	17.35	6.69	62.8
Total respiratory	6859	17.62	8667	22.26	4.64	
Hepatitis	6566	16.87	5594	14.37	−2.50	−14.8
Hepatosis/hepato-dystrophies	1	0.00	618	1.59	1.58	61,700
Peritonitis/perihepatitis	66	0.17	71	0.18	0.01	7.58
Enteritis	38	0.10	137	0.35	0.25	261
Total digestive	6671	17.14	6420	16.49	−0.65	
Nephritis	35	0.09	113	0.29	0.20	223
Nephrosis	163	0.42	224	0.58	0.16	37.4
Cryptorchidism	40	0.10	115	0.30	0.19	188
Total reproductive-urinary	238	0.61	452	1.16	0.55	
Myocarditis	1	0.00	4	0.01	0.01	300
Pericarditis	575	1.48	1059	2.72	1.24	84.2
Total cardio-circulatory	576	1.48	1063	2.73	1.25	
Dermatitis	520	1.34	858	2.20	0.87	65.0
Erysipelas	148	0.38	291	0.75	0.37	96.6
Total tegumentary	668	1.72	1149	2.95	1.24	
Arthritis	0	0.00	0	0.00	0.00	
Muscle colour alteration (PSE/DFD)	0	0.00	4	0.01	0.01	
Oedema/emaciation	3	0.01	4	0.01	0.00	33.3
Total locomotor	3	0.01	8	0.02	0.01	
Jaundice	4	0.01	9	0.02	0.01	125
Abscesses	454	1.17	422	1.08	−0.08	−7.05
Neoplasms / tumours	3	0.01	9	0.02	0.02	200
Biliary or faecal contamination	685	1.76	1161	2.98	1.22	69.5
Trauma	103	0.26	674	1.73	1.47	554
Lymphadenopathy	6	0.02	25	0.06	0.05	317
Splenomegaly	111	0.29	176	0.45	0.17	58.6
Petechial haemorrhages	1	0.00	0	0.00	0.00	−100
Total other	1367	3.51	2476	6.36	2.85	
Tot lesions	16,382		20,235			
Tot Animals	38,929		38,929			
% lesions	42.09		51.98

**Table 4:** Means, standard deviations and percent variation coefficients of lesion detection achieved by contracted veterinarians in the preliminary phase, when they inspected 5000 carcass each (not included in the global database), and the comparison period at the end of the study, after the guidelines were applied

	Preliminary period			Final period			
	mean	st. dev.	v. c.	mean	st. dev.	v. c.	Δ v.c. after training
Pneumonia	8.58	8.88	103.52	4.69	3.83	81.66	−21.85
Pleuropneumonia	10.86	7.21	66.39	17.29	1.78	10.31	−56.08
Hepatitis	17.00	9.10	53.54	14.56	2.81	19.26	−34.28
Hepatosis/hepato-dystrophies	1.72	2.61	151.90	1.36	2.17	159.73	7.83
Peritonitis/perihepatitis	0.55	0.54	97.81	0.20	0.17	83.45	−14.36
Enteritis	0.30	0.27	90.53	0.38	0.22	59.68	−30.84
Nephritis	0.18	0.07	40.29	0.26	0.15	59.44	19.14
Nephrosis	0.16	0.16	97.47	0.55	0.25	45.50	−51.98
Cryptorchidism	0.14	0.03	24.34	0.30	0.06	20.80	−3.55
Myocarditis	0.02	0.02	150.55	0.01	0.02	113.93	−36.62
Pericarditis	2.96	1.02	34.43	2.71	0.50	18.61	−15.82
Dermatitis	0.96	0.67	69.44	1.97	1.11	56.40	−13.04
Erysipelas	0.06	0.09	154.20	0.71	0.68	96.10	−58.10
Arthritis	0.01	0.01	167.33	0.00	0.00	0.00	−167.33
Muscle colour alteration (PSE/DFD)	0.00	0.01	154.92	0.01	0.01	115.72	−39.20
Oedema/emaciation	0.02	0.02	89.57	0.01	0.01	120.94	31.36
Jaundice	0.04	0.03	88.06	0.03	0.03	96.50	8.44
Abscesses	0.74	0.28	38.07	1.09	0.35	31.87	−6.20
Neoplasms / tumours	0.01	0.01	77.46	0.04	0.08	200.00	122.54
Biliary or faecal contamination	2.44	0.90	36.85	3.10	0.88	28.44	−8.41
Trauma	0.56	0.71	125.58	1.58	1.13	71.25	−54.33
Lymphadenopathy	0.14	0.19	143.46	0.06	0.04	62.70	−80.77
Splenomegaly	0.24	0.19	82.18	0.46	0.24	50.97	−31.21
Petechial haemorrhages	0.02	0.02	150.55	0.00	0.00	0.00	−150.55
Total	47.66	19.02	39.91	51.37	5.51	10.73	−29.19

## Discussion

The majority of lesions were at the respiratory level (Table 2). In fact, more than 20% of the animals had pneumonia or pleuropneumonia. This result is not surprising because intensively bred, fat animals nine months in age were inspected. Furthermore, these data are consistent with those coming from international literature. For instance, in a review of post-mortem data in pig slaughterhouses of New Zealand from 2000 to 2010, Neumann et al. [14] found a prevalence of pleurisy, pneumonia and pleuropneumonia of approximately 16%. This prevalence is slightly lower than the one found in the present study, which can be explained by the lower age and weight of their animals at the time of slaughter.

For heavy pigs from Northern Italy, Merialdi et al. [15], found a prevalence of respiratory lesions of up to 40%, which is even higher than the prevalence in the present study. However, the focus of this previous study was different, and the researchers probably included all minimal lung lesions. In the present study, pneumonia was considered only if the lesion (Table 1) intersected a whole lobe. They found a prevalence of milk spot lesions near 10%, while in the present study, the prevalence of hepatic lesions was 16%. Milk spot lesions composed the majority of hepatic lesions in the present study, but the fact that all hepatic lesions were not classified in more detail can explain the difference in results.

According to European Union Regulation (EC) No. 854/2004, erysipelas should be detected ante-mortem, and the slaughtering must be deferred. Nevertheless, erysipelas can be undiagnosed ante-mortem because the typical lesions become evident only after scalding and bristle removal. In this case, swine carcasses affected by erysipelas must either undergo skin removal or be destroyed depending on the disease stage. Occasional cases of erysipelas were recorded during post-mortem inspection, but the number was very low. In all of these cases, the carcasses were destroyed.

No large differences were detected between the three slaughterhouses. In particular, as could be expected due to the homogeneity of the animals, no relevant differences in lesions related to animal health were found. Only a relevant difference in biliary or faecal contamination was found. In particular, one slaughterhouse showed an prevalence of carcass contamination (3.6%) that was much higher than that of the other two slaughterhouses (2.2% and 2.5%). The slaughtering lines of the three plants did not have relevant technological differences. The two slaughterhouses with lower incidences had a visual inspection of carcasses for faecal or biliary contamination, defined as a critical control point in their self-control plan, while the third slaughterhouse did not. This difference probably resulted in the operators paying greater attention during the evisceration phases.

No differences in trauma lesions were found between the slaughterhouses. The relatively low number of cases (2395, 1.03%) shows that the operators pay attention to animal welfare and handling during transportation and ante-mortem care.

Overall, the kidney conditions of the animals were good, and nephritis or nephrosis lesions were detected in less than 0.3% of the cases.

Dermatitis lesions were found in approximately 1.4% of cases. This figure is much lower than the data recorded by Neuman et al. [14], who found mange lesions in 3.6% of the animals. Still, the data can be considered comparable because dermatitis in the present study was recorded only when the lesion involved more than 50% of the whole skin surface (Table 1).

Regarding peritonitis/perihepatitis, enteritis, cryptorchidism, pericarditis, abscesses and splenomegaly, it is almost impossible to compare these data with international literature since these data are scarce.

Myocarditis, arthritis, muscle colour alteration, oedema/emaciation, jaundice, neoplasms/tumours, lymphadenopathy and petechial haemorrhages cannot be considered since their prevalence was lower than 0.1%, and at this level, the relative standard error of the estimate is too high to make reliable conclusions.

### Visual vs. traditional inspections

As a whole, the visual-only inspection showed greater efficiency than the traditional inspection in detecting lesions (Table 3). In fact, the visual-only inspection detected lesions in 52% of the animals, while the traditional inspection detected lesions in only 42% of the animals. There was a large difference in the sensitivity in pneumonia and pleuropneumonia detection probably because official veterinarians performing traditional inspections did not undergo training for lesion classification before the trial. As a matter of fact, if we consider respiratory lesions (pneumonia and pleuropneumonia) together, the difference is much lower and not statistically relevant. In synthesis, comparable numbers of respiratory diseases were detected by both systems, but the lesion classifications were different.

In addition, the difference in hepatitis detection ability was not statistically relevant, but it is not surprising that traditional liver palpation leads to more sensitivity in this area.

As a whole, almost the same sensitivity was noticed in detecting lesions in red and green offal, while visual-only inspection showed greater sensitivity in detecting lesions on the carcass. The slaughtering line was working at 380 pigs per hour, meaning that there was less than 10 s to perform a whole post-mortem inspection. If the veterinarian had to perform invasive actions, the time available for looking at the whole carcass was probably too short.

**Fig. 2:**
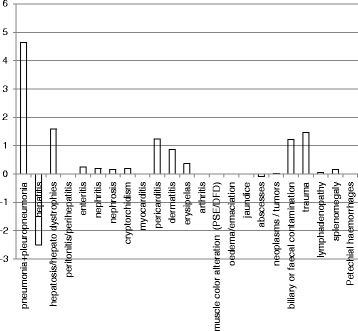
Percent differences between visual and traditional inspections (positive values represent greater sensitivity of visual inspection, and negative values lower represent lower sensitivity)

The analytical results agree with an assessment of risk associated with changes in meat inspections conducted by the Danish Agriculture and Food Council in 2014 [16], which found higher sensitivity for visual inspections than traditional inspections. Hill in 2013 [17], Mousing in 1997 [18] and Blagojevich in 2015 [19] also stressed that switching to visual inspection in pigs does not imply an increase in risk, even if the pigs are raised outdoors. Figure 2 graphically represents the differences between the two inspection systems.

### Pre- and post-training evaluation of veterinarians

To conduct this analysis, it was postulated that on a very large number of inspected animals, each operator should obtain the same mean data. This approach was only possible in field conditions. In such a scenario, the deviation from the median is a good parameter to define how good the inspector is compared to other colleagues. Obviously, such a system is most reliable and meaningful for the most common lesions, and it is not reliable for more “exotic” lesions.

After setting guidelines and training, a generally low variation was achieved at the end of the study period, as shown by a decrease in the variation coefficient of almost every lesion category (Table 4). The decrease was present in common lesions and in the total number of lesions, showing that training is crucial to obtain homogenous judgements by veterinarians. This approach was not successful for detecting and classifying every lesion (e.g., hepatosis and nephritis), but one of the functions of such an instrument is the ability to address future training actions.

### Official vs. contracted veterinarians

The same principle used for evaluating pre- and post-training performance was adopted to compare the performance of official and contracted veterinarians. Following this principle, the official veterinarians that inspected a low number of animals were excluded from this analysis. The classification and recording of lesions can be extremely useful because these data can be used for epidemiological purposes, for farming suggestions and even for farm classification. However, such a system can be effective only if the inspector’s judgements are repeatable and reliable. As much as possible, the inspections have to be independent of the individuals conducting the inspections. Moreover, these judgements have extremely important economic relevance since different condemnation rates of single organs or whole carcasses imply different costs both for slaughterers and for famers.

From the data in Table 5, it is clear that the trained contracted veterinarians achieved a globally lower variability than the official colleagues. The fact that the official veterinarians were not trained to apply the guidelines can easily explain the difference. The data demonstrate that it is essential to reach a high level of standardisation, which can be achieved only through the adoption of strict operative guidelines and training veterinarians to adopt and follow these guidelines. The training should be aimed towards reaching a lower variability in judgement by understanding and following the guidelines.

**Table 5:** Means, standard deviations and percent variation coefficients of lesion detection achieved by the official veterinarians performing traditional inspection and by the contracted veterinaries performing visual-only inspection in the comparison period

	Official veterinarians			Contracted veterinarians			
	mean	st. dev.	v. c.	mean	st. dev.	v. c.	δ over official
Pneumonia	5.82	4.39	75.57	4.69	3.83	81.66	6.09
Pleuropneumonia	10.70	2.86	26.76	17.29	1.78	10.31	−16.45
Hepatitis	14.96	8.80	58.83	14.56	2.81	19.26	−39.56
Hepatosis/hepato-dystrophies	0.00	0.00	300.00	1.36	2.17	159.73	−140.27
Peritonitis/perihepatitis	0.11	0.26	239.75	0.20	0.17	83.45	−156.30
Enteritis	0.04	0.07	181.11	0.38	0.22	59.68	−121.43
Nephritis	0.05	0.06	123.58	0.26	0.15	59.44	−64.15
Nephrosis	0.21	0.32	152.40	0.55	0.25	45.50	−106.91
Cryptorchidism	0.06	0.09	161.92	0.30	0.06	20.80	−141.13
Myocarditis	0.00	0.00	300.00	0.01	0.02	113.93	−186.07
Pericarditis	1.81	1.20	66.13	2.71	0.50	18.61	−47.52
Dermatitis	0.80	1.65	205.77	1.97	1.11	56.40	−149.37
Erysipelas	0.39	0.32	82.58	0.71	0.68	96.10	13.52
Arthritis	0.00	0.00	0.00	0.00	0.00	0.00	0.00
Muscle colour alteration (PSE/DFD)	0.00	0.00	0.00	0.01	0.01	115.72	115.72
Oedema/emaciation	0.01	0.01	217.58	0.01	0.01	120.94	−96.64
Jaundice	0.01	0.01	198.29	0.03	0.03	96.50	−101.79
Abscesses	0.97	0.55	56.44	1.09	0.35	31.87	−24.57
Neoplasms / tumours	0.02	0.05	254.11	0.04	0.08	200.00	−54.11
Biliary or faecal contamination	1.96	0.64	32.94	3.10	0.88	28.44	−4.50
Trauma	0.10	0.21	205.51	1.58	1.13	71.25	−134.26
Lymphadenopathy	0.01	0.02	198.62	0.06	0.04	62.70	−135.92
Splenomegaly	0.14	0.19	135.20	0.46	0.24	50.97	−84.24
Petechial haemorrhages	0.00	0.00	300.00	0.00	0.00	0.00	−300.00
Total	38.15	16.30	42.71	51.37	5.51	10.73	−31.98

Nine official veterinarians conducted inspections during the study, but two of these veterinarians were excluded in this evaluation since they inspected less than 1000 carcasses

## Conclusions

The data derived from local projects on post-mortem lesions in slaughterhouses in Northern Italy were not homogenous and comparable.

For the first time, a classification of lesions was developed and shared with the Ministry of Health and the two most productive regions in the swine sector. Moreover, a relevant dataset of these lesions and instruments able to further expand this database were built.

In industrial high-speed slaughtering lines of pigs, visual inspection was shown to be comparable to traditional inspection and was even more sensitive for some lesions.

Post-mortem inspection is a human judgement and is therefore prone to large error. To minimize error and to achieve a high level of standardization, it is necessary to develop operative guidelines. In addition, training the operators involved is crucial for obtaining consistent data. Only with reliable data can post-mortem inspection reports be used for several purposes, such as epidemiological studies or the classification of farms based on risk. It is therefore important to have the same classification and guidelines, and the veterinarians involved in meat inspection should undergo continuous education.
